# Ego depletion interferes with rule-defined category learning but not non-rule-defined category learning

**DOI:** 10.3389/fpsyg.2015.00035

**Published:** 2015-01-29

**Authors:** John P. Minda, Rahel Rabi

**Affiliations:** Department of Psychology, University of Western Ontario, London, ONCanada

**Keywords:** categorization, self regulation, ego depletion, COVIS, multiple systems, category learning

## Abstract

Considerable research on category learning has suggested that many cognitive and environmental factors can have a differential effect on the learning of rule-defined (RD) categories as opposed to the learning of non-rule-defined (NRD) categories. Prior research has also suggested that ego depletion can temporarily reduce the capacity for executive functioning and cognitive flexibility. The present study examined whether temporarily reducing participants’ executive functioning via a resource depletion manipulation would differentially impact RD and NRD category learning. Participants were either asked to write a story with no restrictions (the control condition), or without using two common letters (the ego depletion condition). Participants were then asked to learn either a set of RD categories or a set of NRD categories. Resource depleted participants performed more poorly than controls on the RD task, but did not differ from controls on the NRD task, suggesting that self regulatory resources are required for successful RD category learning. These results lend support to multiple systems theories and clarify the role of self-regulatory resources within this theory.

## INTRODUCTION

Learning to classify things is a fundamental cognitive process. A variety of theoretical approaches have been developed to explain how new categories are acquired and how categories are represented in the mind as concepts. One prominent and well-studied class of theories assumes that new categories are acquired by means of at least two broadly defined cognitive systems. The COVIS model (COmpetition between Verbal and Implicit Systems) is a well-known version of these so-called multiple systems theories (Ashby et al., 1998; Maddox and Ashby, 2004; Minda and Miles, 2010; Miles and Minda, 2011). COVIS assumes that an explicit, verbally mediated system relies on verbal working memory and executive functions to learn categories that can be defined by an easily verbalizable rule. An implicit, non-verbal system relies on associative learning mechanisms to learn categories that lack an easily verbalizable rule.

**FIGURE 1:**
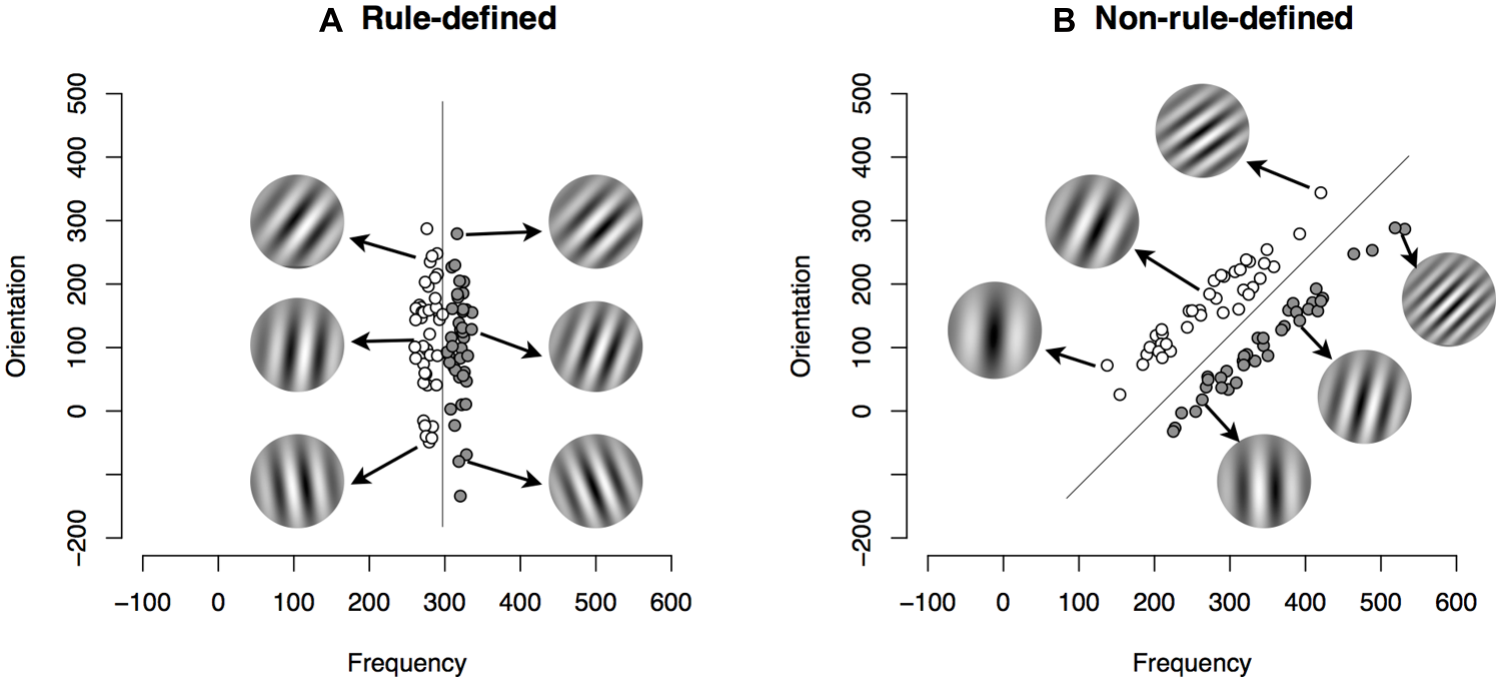
**(A)** Category structure for the rule-defined (RD) category set. Each light circle represents a stimulus from Category A and each dark circle represents a stimulus from Category B. The line shows the optimal boundary between the stimuli. The six sine-wave gratings demonstrate examples of the actual stimuli seen by participants. **(B)** Category structure for the non-rule-defined (NRD) information-integration category set.

As an example of the difference between rule-defined (RD) and non-rule- defined (NRD) categories, consider the stimuli shown in **Figure 1**. These stimuli vary along two dimensions, the tilt of the alternating light and dark bands, as well as the spatial frequency of the alternating light and dark bands. The RD set shown in **Figure 1A** would require learners to find a single-dimensional rule (spatial frequency in this case) and to simultaneously inhibit responding to another dimension (orientation). In **Figure 1B**, both orientation and frequency are needed to learn the NRD categories and there is no easily verbalizable rule to classify the stimuli.

### EMPIRICAL EVIDENCE FOR TWO SYSTEMS

Considerable research over the last decade has established the characteristics and boundary conditions of each of the systems, and has shown how various cognitive, behavioral, and contextual constraints affect each system. For example, when participants are asked to learn a set of RD categories (like those in **Figure 1**), their performance on the task is reduced if they learn the categories while performing a concurrent task that interferes with working memory or executive function, such as a Stroop task or a digit span task (Zeithamova and Maddox, 2006; Minda et al., 2008). The same concurrent task has little or no effect on NRD categories. If participants are asked to learn NRD categories with a task that interferes with procedural learning, such as switching the location of the response keys or delaying onset of feedback after the response, performance on NRD tasks is reduced whereas performance on RD tasks is not affected (Maddox et al., 2003, 2009; Maddox and Ing, 2005).

Imaging and cognitive neuroscience research has clarified the contributions of several brain areas related to COVIS and category learning (Reber et al., 1998, 2003; Ashby and Maddox, 2005; Ashby and Ennis, 2006; Nomura et al., 2006; Nomura and Reber, 2008; Seger and Miller, 2010). Broadly speaking, the prefrontal cortex plays a critical role in carrying out the hypothesis testing and rule selection functions of the verbal/explicit system. The prefrontal cortex also seems to play a role in mediating between systems. Imaging research also suggests a primary role of the striatal cortex and the tail of the caudate in the implicit systems. Both systems assume a central role for dopamine with respect to enhancing the flexibility in the verbal system, as well as mediating feedback in the implicit system (Ashby et al., 1999, 2007).

Other research has examined the learning of RD and NRD categories at different developmental stages. For example, young children seem to have difficulty learning RD categories relative to adults, because the underlying neural systems that mediate the explicit/verbal system develop more slowly than the systems that mediate the implicit system (Minda et al., 2008; Huang-Pollock et al., 2011; Rabi and Minda, 2014; van Bers et al., 2014). Some research argues that working memory capacity can explain many of these effects within a single system (Lewandowsky et al., 2012). However, other work also suggests a role for inhibitory control in managing RD category learning. A recent, fairly extensive study of children ranging from age 4 through adolescence, showed that RD categorization performance improved with age. A larger working memory capacity as well as strong inhibitory control abilities were associated with increased RD categorization performance (Rabi and Minda, 2014).

This developmental work is consistent with comparative research showing that while many species lack the cognitive and neurobiological requirements for COVIS’s explicit system, other species can learn single dimensional rules. Pigeons and rats lack these functions and show a pattern of performance that indicates a primary reliance on similarity and associative leaning that is consistent with the procedural system (Smith et al., 2011; Vermaercke et al., 2014). However, many non-human primates possess the cognitive precursors to the explicit system, such as a tendency to dimensionalize stimuli. Primates show the dissociation between the two systems, though often rely too heavily on a single dimension and seem to fail to learn more complicated rules. (Smith et al., 2004, 2010, 2011, 2012; Smith, 2013).

Finally, some research has examined contextual effects like mood. Nadler et al. (2010) induced a positive, neutral, or negative mood in their participants, and then asked them to learn either a RD or a NRD category set. Positive mood improved performance on the RD task relative to the neutral or negative conditions. But the same positive mood condition did not improve performance on the NRD task. Nadler et al. (2010) did show that positive mood affected strategy selection in the NRD task, by allowing participants to adopt a more adaptive, information integration strategy. This work suggests a link between positive mood, cognitive flexibility, and performance on RD tasks. This reinforces the notion that the explicit/verbal system relies on working memory and executive function, benefitting from improvements in cognitive flexibility.

Taken collectively, this research paints a picture of an explicit/verbal system that relies on working memory and executive functions and uses these cognitive functions to acquire rules and learn new categories. The empirical research reviewed suggests that cognitive and contextual variables that interfere with executive functions should have a deleterious effect on learning RD categories but not learning NRD categories. At the same time, cognitive and contextual variables that might enhance executive function or cognitive flexibility, should have a facilitatory effect when learning RD categories, but not when learning NRD categories.

### EGO DEPLETION

Much of the empirical research with COVIS has examined cognitive and contextual effects that are concurrent with learning (e.g., dual tasks, developmental interference, etc.). One exception is the mood induction paradigm in which positive mood improved performance on learning an RD category set. A possible explanation is that the mood induction had a lasting positive effect on cognitive flexibility that facilitated hypothesis testing during the rule acquisition task. An intriguing possibility is that other prior tasks may also have a lasting negative effect on executive function and cognitive flexibility that will interfere with RD learning, but have little or no effect on NRD learning.

Recent literature on a phenomenon known as “ego depletion” provides one way to test this hypothesis. The core idea, taken from Baumeister et al. (2007) is that self regulation is a finite resource. Just as a muscle tires from continuous exertion, so too does the self-regulation process tire. Maintaining performance in a demanding cognitive task can deplete resources, and these depleted resources are known to have a detrimental effect on subsequent tasks that depend on them. Baumeister (2014) used the term “ego-depletion” as an homage to Freud though they stress that their theory does not bear a theoretical resemblance to Freud’s theories. An extensive literature provides some idea as to how ego depletion works, under what circumstances, and why.

In one of the first studies of ego depletion, Baumeister et al. (1998) found that performing an act of self-regulation affects performance on a subsequent executive function task, suggesting that the two types of tasks share resources. For example, they found that when participants forced themselves to eat radishes instead of tempting chocolates, they displayed reduced persistence on a subsequent puzzle-solving task compared to participants who did not exert self-control over eating. When asked to suppress emotional distress, participants displayed impaired subsequent performance at solving anagrams. A study by Schmeichel (2007) showed that participants who were asked to engage in a task that depleted their cognitive regulation resources, such as regulating their emotions, controlling their attention, or taking a working memory test performed more poorly on subsequent tests of working memory span and inhibitory control. Finally, depletion also influences decision-making abilities. Depleted participants make poorer decisions, and fail to take in account decision alternatives as well as control individuals. Depleted individuals also tend to depend more heavily on heuristics and often fail to weigh all of their options carefully (Masicampo and Baumeister, 2008).

Other research has attempted to explain the biology of ego depletion. Just as engaging in self-regulatory behavior depletes resources, rest or refueling counteracts those effects. For example, Gailliot et al. (2007) asked participants to engage in a variety of self-regulatory behaviors, such as ignoring text on a visual display, suppressing stereotypes, or engaging in a demanding Stroop task. Gailliot et al. (2007) found that across these tasks, exerting self-control used up a relatively large amount of measurable glucose (as measured via blood glucose levels pre and post test). Interestingly, replenishing blood glucose with a sugary drink counteracted ego depletion effects. In other words, the ego depletion task depleted resources, but drinking the energy drink restored them.

Finally, research using sleep deprivation as a comparison, has shown that ego depletion is not the same as general fatigue (Vohs et al., 2011). Sleep deprived participants suffered from fatigue and did not display the ego depletion effects. The authors argue that, unlike general fatigue, ego depletion is the “exhaustion of the inner energy that modulates unwanted responses.”

The implications for category learning and multiple systems specifically should be clear. Our earlier work, along with other research on COVIS, indicates a strong role for executive functions in the explicit/verbal system that is used to acquire RD categories. If participants are asked to engage in an ego depletion task and then asked to learn either a set of RD categories, or a set of NRD categories, we predict that the ego depletion task should reduce performance relative to controls on RD categories, but should have little or no effect on NRD categories. This result would add to an already rich body of research suggesting that disrupting executive functions affects category learning by the explicit/verbal system and not the procedural category learning system. As such, we predict that this result offers additional support for COVIS as an explanatory system. In addition, this result also adds to the literature on ego depletion and extends the paradigm to include classification and categorization as cognitive processes that may be affected by the reduction in self-regulatory resources.

### EXPERIMENT

We designed an experiment to test the prediction that performing an ego depletion task would reduce performance relative to a control task when participants are asked to learn a set of RD categories. However, performing the ego depletion task should have no effect or little effect when participants are asked to learn a set of NRD categories. We chose an ego depletion task from Schmeichel (2007) in which participants are asked to write a story without using two common letters, “a,” and “n.” Control participants were not given the letter restriction. Schmeichel (2007) found that participants in the letter restriction task were required to constantly inhibit the tendency to use these letters and thus showed reduced performance on subsequent tasks in line with the ego depletion effect.

We adopted a category set common in the literature that distinguishes between rule acquisition and associative learning (Maddox et al., 2006; Nadler et al., 2010). **Figure 1** shows an example of our stimuli, which are explained in greater detail below. The stimuli vary along two dimensions, the tilt of the alternating light and dark bands, as well as the spatial frequency of the alternating light and dark bands. The RD set shown in **Figure 1A** required learners to find a single-dimensional rule (spatial frequency in this case) and to simultaneously inhibit responding to another dimension (orientation). The NRD categories set did not have a verbalizable rule and did not require the inhibition of any one dimension. In **Figure 1B**, both orientation and frequency are needed, and there is no easily verbalizable rule to classify the stimuli.

We relied on a 2 × 2 design. Half of our participants were given the ego depletion task, and half were given the control task. Half of our participants were given the RD categories to learn, and the other half were given the NRD categories to learn. As stated above, we predict that performing the ego depletion task will reduce performance on the RD categories, but will have little or no effect on performance on the NRD categories.

## MATERIALS AND METHODS

### PARTICIPANTS

Participants were 77 University students (25 males and 52 females) from the University of Western Ontario. Participants were recruited from an introductory psychology class and were given course credit for their participation. Participants were randomly assigned to either the ego depletion condition or the control condition. Three participants ^1^ in the ego depletion condition were excluded from data analysis because they made too many errors (they used the restricted letters) on the story-writing task, suggesting that they were not completing the task in the manner required to induce a state of resource depletion. There were 20 participants in the RD control group, 19 in the RD ego depletion group, 18 in the NRD control group, and 17 in the NRD ego depletion group.

### MATERIALS

The ego depletion task was adapted from Schmeichel (2007). Participants were given 10 min to write a story describing a trip they had taken recently. Participants in the ego depletion condition were instructed to write their story without using the letters “a” and “n.” To complete this task, some degree of self-regulation is required to inhibit the use of two frequently used letters, as well as the possible recruitment of other executive resources to search for alternative words. Participants in the control condition were not given any restrictions, and so participants did not have to inhibit any writing tendencies. Following the story-writing task, participants were administered a mood measure, the Positive and Negative Affect Schedule, or PANAS (Watson et al., 1988), before completing the category learning task.

For the category learning task, subjects classified sine-wave gratings that varied in spatial frequency and orientation. 80 stimuli were generated for each category set (Ashby and Gott, 1988; Zeithamova and Maddox, 2006), with 40 stimuli in each category. We randomly sampled 40 values from a multivariate normal distribution described by each category’s parameters (shown in **Table 1**). The resulting category structures for RD and NRD category sets are illustrated in **Figure 1**. We then used the PsychoPy package (Peirce, 2007) to generate sine wave gratings corresponding to each coordinate sampled from the distributions above. For both category sets sine wave grating frequency was calculated as *f* = 0.25 + (*x_f_*/50) cycles per stimulus and orientation was calculated as *o* = *x_o_* × (π/20) degrees.

**Table 1:** Distribution parameters for rule-defined (RD) and non-rule-defined (NRD) category sets.

Category structure	μ_f_	μ_o_	σ_f_^2^	σ_o_^2^	*cov_f,o_*
**RD categories**
Categorie A	280	125	75	9,000	0
Categorie B	320	125	75	9,000	0
**NRD categories**
Categorie A	268	157	4,538	4,538	4,351
Categorie B	332	93	4,538	4,538	4,351

*Dimensions are in arbitrary units; see the Materials section for a description of the scaling factors. The subscripted letters *o* and *f* refer to orientation and frequency, respectively*.

### PROCEDURE

Participants were tested in groups of one to four participants at a time. Participants were randomly assigned to either the ego depletion group or the control group, and all participants that were run simultaneously were assigned to the same condition. Participants were also randomly assigned to learn either the RD categories or the NRD categories, however, we did not assign the same category structure to all participants who were run during the same session.

Participants were seated at a desk with a monitor, keyboard, and Apple Mac mini computer. After completing an initial consent form, participants wrote a story describing a trip they had taken recently. Participants in the ego depletion group were asked to write a story without using the letters “a” or “n.” Participants in the control group were given no restrictions on which letters to use. The experimenter stopped all participants after 10 min of writing and gave them the PANAS questionnaire, as a general measure of their current mood. We included this as a possible manipulation check, and because prior work suggested that positive mood was a strong enhancer of performance on the RD categorization task (Nadler et al., 2010).

Participants next completed the categorization task. They were given initial instruction that they would be seeing a “crystal ball” on the screen and their job was to determine whether that crystal ball belonged to the blue wizard category or the green wizard category. They were instructed to press the key labeled “green” to make a green wizard response and to press the key labeled “blue” to make a blue wizard response. Participants were told they would receive feedback after every response, and that they should use this feedback to help them learn to make as many correct responses as possible.

**FIGURE 2:**
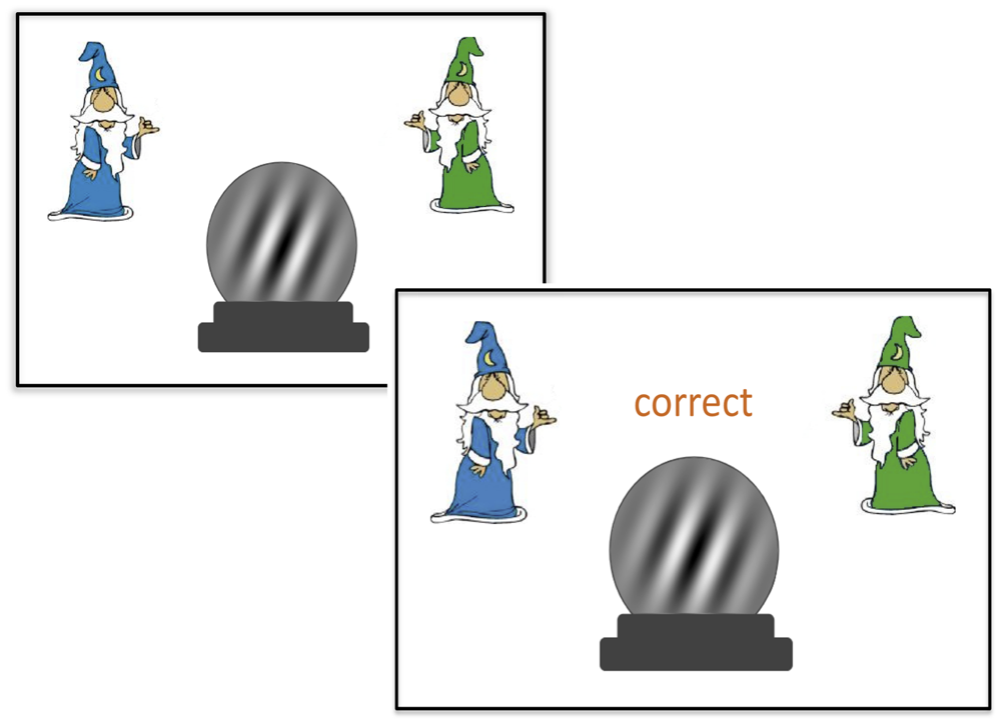
**An example of a correct trial in the categorization task.** Feedback was presented after each response (“correct” or “incorrect”).

Participants were presented with four blocks of the 80 stimuli, 320 trials in total. Within a block, the order of presentation of all 80 stimuli in the category set was randomized. On each trial, participants saw the crystal ball in the center of the screen and a blue wizard and green wizard in the upper left and upper right corner of the screen. Upon making a response, feedback was delivered in the space between the stimulus and the two wizards as shown in **Figure 2**. The word “correct” or “incorrect” was presented after each response.

Upon completion of the category learning task, participants completed a follow-up questionnaire by rating the difficulty of the initial story writing task and also the difficulty of the categorization task (on scales from 1 = *not at all difficult* to 7 = *very difficult)*.

## RESULTS

### MANIPULATION CHECK

As a manipulation check, we first examined the self-reported difficulty on the story-writing task. Not surprisingly, participants in the ego depletion condition rated the task as being significantly more difficult (*M* = 5.42, *SD* = 1.13) than participants in the control condition (*M* = 1.61, *SD* = 1.00), *F*(1,72) = 236.29, *p* < 0.001.

### CATEGORY LEARNING PERFORMANCE AS A FUNCTION OF CATEGORY TYPE, CONDITION, AND LEARNING BLOCK

A 4 (learning block) × 2 (category type: RD, NRD) × 2 (condition: ego depletion, control) mixed analysis of variance was conducted. Results revealed a significant main effect of category type, *F*(1,70) = 25.14, *p* < 0.001, partial η^2^ = 0.26. As well as a main effect of block, *F*(2.4,169) = 41.43, *p* < 0.001 (Greenhouse–Geisser corrected), partial η^2^ = 0.37, demonstrating that categorization performance improved over time. A linear trend analysis supported this conclusion (linear contrast, *F*(1,70) = 69.50, *p* < 0.001). There was no main effect of condition, *F*(1,70) = 3.04, *p* = 0.086, partial η^2^ = 0.04. Aside from the block by category type interaction, *F*(2.4,169) = 6.14, *p* = 0.001 (Greenhouse–Geisser corrected), partial η^2^ = 0.08, no other interactions reached significance (all *P*s > 0.13) ^2^. In order to further explore the block by category type interaction, we conducted two separate analyses of variance (one for the RD category and one for the NRD category).

### PERFORMANCE ON THE RD TASK

**FIGURE 3:**
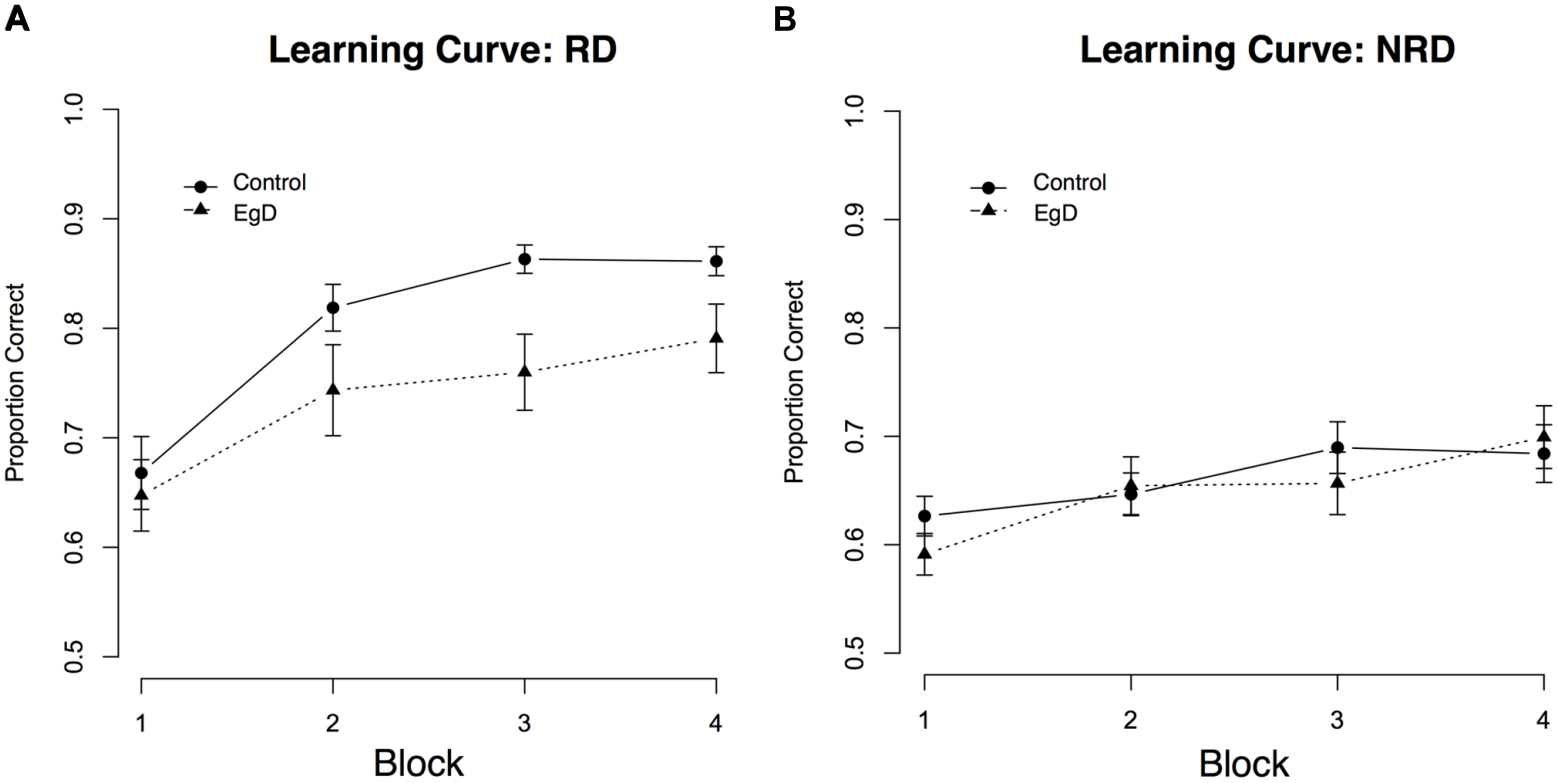
**Average proportion of correct responses to stimuli in the ego depletion and control condition as a function of trial block.** Participants were tested on either the RD category set **(A)** or the NRD category set **(B)**. Error bars denote SE of the mean.

We calculated the average proportion correct at each block for participants in the ego depletion condition and participants in the control condition. The averages are shown in **Figure 3A**, and indicate that participants in the ego depletion condition learned the categories less well than participants in the control condition. We entered all the proportion correct data into a 4 × 2 ANOVA with block (1–4) as a within subjects factor and condition (ego depletion and control) as a between subjects factor. The ANOVA revealed a marginally significant main effect of condition, *F*(1,37) = 3.74, *p* = 0.06, and a significant main effect of block, *F*(2.3,84) = 37.38, *p* < 0.001 [Greenhouse–Geisser corrected; linear contrast: *F*(1,37) = 53.88, *p* < 0.001]. There was no significant interaction between block and condition, *F*(2.3,84) = 1.89, *p* = 0.15 [Greenhouse–Geisser corrected].

While the block by condition interaction was not significant, visual inspection of the RD learning curve illustrates that performance differences become more apparent later in learning. As can be seen in **Figure 3A**, both ego depletion participants and controls performed similarly early in learning (blocks 1 and 2), most likely because they were all getting acquainted with the task. That is, performance is generally low and does not differ between conditions. Later in learning (blocks 3 and 4), a more stable categorization performance becomes evident and participants begin to apply a more consistent strategy. To further examine RD block-wise performance across conditions, Bonferroni corrected *post hoc* tests were conducted. Early in learning, the categorization performance of participants in the ego depletion condition did not significantly differ from controls in both block 1 (*p* = 0.66) and block 2 (*p* = 0.11). However, later in learning, performance differences emerged, with controls significantly outperforming ego depletion participants in both block 3 (*p* = 0.007) and block 4 (*p* = 0.042).

### PERFORMANCE ON THE NRD TASK

We calculated the average proportion correct at each block for participants in the ego depletion condition and participants in the control condition. The averages are shown in **Figure 3B**, and indicate that participants in the ego depletion condition learned the categories about as well (or as poorly) as participants in the control condition. We entered the proportion correct data into a 4 × 2 ANOVA with block (1–4) as a within subject factor and condition (ego depletion and control) as a between subject factor. The ANOVA revealed no main effect of condition, *F*(1,33) = 0.17, *p* = 0.68, and a significant main effect of block, *F*(2.5,81) = 9.13, *p* < 0.001 [Greenhouse–Geisser corrected; linear contrast: *F*(1,33) = 19.26, *p* < 0.001]. There was no significant interaction between block and condition, *F*(2.5,81) = 1.26, *p* = 0.29 [Greenhouse–Geisser corrected].

In line with these findings, Bonferroni *post hoc* tests revealed no difference in NRD categorization performance between ego depletion participants and controls across any of the learning blocks (all *P*s > 0.19).

### RELATIONSHIP WITH AFFECT

We examined the relationship between performance on the PANAS scale and the experimental condition. Participants in the ego depletion condition (*M* = 2.62, *SD* = 0.80) reported positive affect that was roughly equivalent to participants in the control condition (*M* = 2.51, *SD* = 0.66), *F*(1,72) = 0.37, *p* = 0.55. However, subjects in the ego depletion condition (*M* = 1.71, *SD* = 0.68) were just slightly more negative than subjects in the control condition (*M* = 1.44, *SD* = 0.39), *F*(1,72) = 4.44, *p* = 0.04.

### SUBJECTIVE EVALUATION OF THE CATEGORIZATION TASK

We also examined the perceived difficulty of the category learning task, in order to assess the possibility that the ego depletion task somehow altered the perception of how difficult the subsequent task was. Among participants completing the RD categorization task, there was no significant difference in the self-rated difficulty of the task between ego depletion participants (*M* = 3.53, *SD* = 1.78) and controls (*M* = 3.35, *SD* = 1.18), *F*(1,37) = 0.135, *p* = 0.72. Among participants completing the NRD categorization task, there was also no significant difference in the self-rated difficulty of the task between ego depletion participants (*M* = 5.06, *SD* = 1.56) and controls (*M* = 4.61, *SD* = 1.46), *F*(1,33) = 0.77, *p* = 0.39.

### COMPUTATIONAL MODELING

For insights into the response strategies used by our participants, we fit a set of decision bound models to each block of each subject’s data (for details see Ashby and Maddox, 1992; Maddox and Ashby, 1993; Miles et al., 2014; Rabi and Minda, 2014). One class of models assumed that each subject’s performance was based on a single-dimensional rule with a fixed intercept and a version with the intercept as a free parameter. We used both an optimal version that based the rule on the spatial frequency and a non-optimal version that based the rule on the orientation dimension. Another class of models assumed that performance was based on the two-dimensional, information-integration boundary (we used an optimal version with a fixed intercept and slope, a version with a fixed slope, and a version with a freely varying slope and intercept). Finally, we fit two guessing models, that assumed no dimensional strategy (one assumed that participants randomly responded *A* or *B* with equal probability for each response and the other assumed unequal probability). We fit these models to each subject’s data by maximizing the log likelihood. Model comparisons were carried out with the AIC index, which penalizes a model for the number of free parameters (Ashby and Maddox, 1992).

**FIGURE 4:**
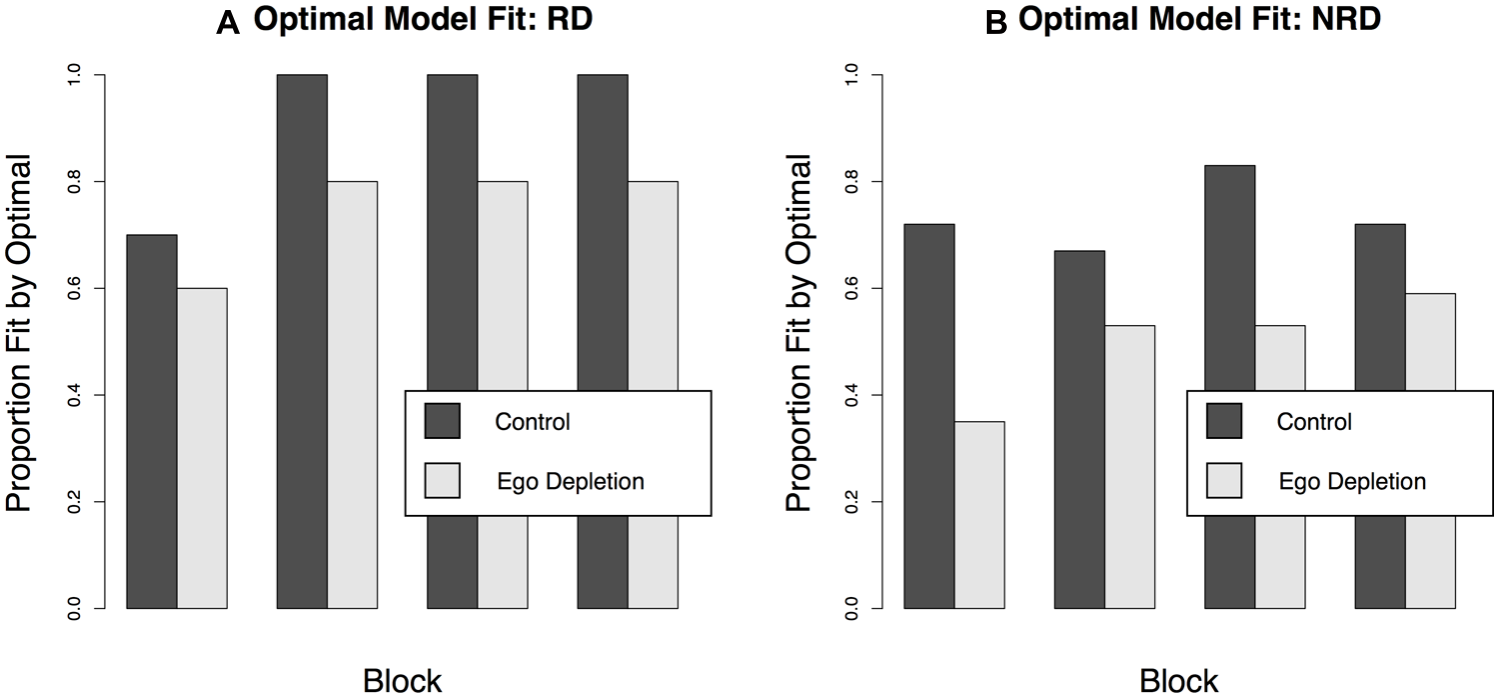
**The proportion of subjects, by block, whose data was fit by the optimal model. (A)** shows the data from the participants who learned the RD categories. The optimal model was the single dimensional rule based on frequency. **(B)** shows the data from participants who learned the NRD categories. The optimal rule was the two dimensional, information integration model.

The proportion of subjects who were best fit by each model is shown in **Table 2**. In general, most participants that learned the RD categories were best fit by the model that assumed the optimal frequency-based model. Participants who learned the NRD categories were fit best by the model that assumed the information integration strategy. **Figure 4** shows the proportion of subjects who were fit best by the optimal model for the category set that they learned as a function of ego depletion condition. Panel A shows that participants in the control condition were generally fit by the optimal rule model. In blocks two, three, and four, all of the participants who learned RD categories in the control condition were best fit by this optimal RD model. In contrast, participants in the ego depletion condition were less likely to be fit by the optimal model. Panel B shows a similar pattern for participants who learned the NRD categories. In this case, the optimal model was the information integration model. Participants who learned in the control condition were more likely to be fit by this model than participants who learned in the ego depletion condition.

**Table 2:** Proportion of subjects fit by each class of decision bound models.

Model?								
**Category**	**Guess**		**II**		**Freq.**		**Ori.**	
	**Cont.**	**EgD**	**Cont.**	**EgD**	**Cont.**	**EgD**	**Cont.**	**EgD**
**RD**
Block 1	0.25	0.37	0.05	0.05	**0.7**	**0.58**	0.00	0.00
Block 2	0.00	0.16	0.00	0.00	**1**	**0.84**	0.00	0.00
Block 3	0.00	0.16	0.00	0.05	**1**	**0.79**	0.00	0.00
Block 4	0.00	0.16	0.00	0.00	**1**	**0.84**	0.00	0.00
**NRD**
Block 1	0.17	0.47	**0.72**	**0.35**	0.00	0.06	0.11	0.12
Block 2	0.17	0.24	**0.67**	**0.53**	0.00	0.12	0.17	0.12
Block 3	0.06	0.29	**0.83**	**0.53**	0.06	0.12	0.06	0.06
Block 4	0.28	0.12	**0.72**	**0.59**	0.00	0.00	0.00	0.29

*Guess, A guessing model; II, The Informational Integration model; “Freq.,” the single-dimensional rule based on the frequency. “Ori.,” the single-dimensional rule based on the orientation. RD, rule defined category set; NRD, non-rule-defined category set; Cont., control condition; and EgD, ego depletion condition. The optimal model for each category set is shown in **bold***.

## DISCUSSION

### SUMMARY OF RESULTS

The data we presented examines a novel prediction of the COVIS model of category learning. COVIS assumes a role for executive function and inhibitory control when learning RD categories but not when learning NRD categories. As a result, any condition that compromises executive function and inhibitory control is likely to result in diminished performance when learning RD categories but not when learning NRD categories. We carried out an experiment to test this hypothesis. Our initial prediction was that participants performing the ego depletion task would show reduced performance when learning RD categories relative to controls. Furthermore, we did not predict an effect of the ego depletion task when participants were asked to learn NRD categories. Our predictions were confirmed: participants in the ego depletion condition did not learn the RD categories as well as control participants.

Consistent with COVIS, which assumes that executive resources and inhibitory control facilitate the hypothesis testing aspect of rule acquisition, participants who were asked to do the story writing task in the ego depletion condition reported difficulty with the task, a slightly elevated negative mood, and showed reduced performance when learning RD categories. This suggests that the self-regulatory and executive resources required by the explicit/verbal system of COVIS are sensitive to the ego depletion effect.

The modeling results suggest a more nuanced view. Consistent with the category learning data, and with the predictions of COVIS, participants who learned the RD categories in the control condition had little trouble adopting an optimal rule based strategy. Participants who learned these categories after completing the ego depletion task were less likely to adopt this optimal rule-based strategy. As a result, there was a clear effect of the ego depletion task on category learning performance. However, we found a similar pattern in the modeling results of participants who learned the NRD categories. Although there was no effect of ego depletion on category learning performance, there seemed to be an effect on strategy use. Participants who learned the NRD categories after completing the ego depletion task were less likely to adopt the optimal information integration strategy. This effect is consistent with earlier research using cognitive tasks (Miles et al., 2014).

The best explanation for this pattern of results is that inhibitory processes were affected by the ego depletion manipulation, and that this reduction in inhibitory processes had a differential effect or RD and NRD category learning. For the RD categories, the reduction of inhibitory processes resulted in fewer participants finding the correct rule, which also reduced overall performance. For the NRD categories, the reduction of inhibitory processes resulted in fewer participants being able to switch away from the initial bias for a rule, and so fewer were able to transition into the correct information integration strategy. However, this slower transition may not produce an observable effect on performance because the NRD categories are learned more gradually by COVIS’s implicit system. This pattern of results is also consistent with COVIS, and Nadler et al. (2010) noted a similar pattern with the effects of mood on the learning of NRD categories. As well, other research has found that an intermittent interference task affects the operation of the explicit system but a continuous interference task disrupts the transition from the explicit system to the implicit system when participants are learning NRD categories (Miles et al., 2014). Others have observed the same kind of effect in aging, where older participants struggle with the transition from rule-based responding to an information-integration based responding when learning NRD categories (Maddox et al., 2010).

### THE ROLE OF INHIBITORY PROCESSES

These results point to the cognitive overlap between the inhibitory process involved in the story writing task (inhibiting the activation of words with “a” or “n”) and the inhibitory process involved in learning the rule (inhibiting the response to incorrect rules). In order for ego depletion to work as described by Baumeister et al. (2007), both the ego depletion task and the subsequent task need to rely on the same kind of self-regulatory process. Research on ego depletion has been fairly consistent on the role of inhibition as one of those behaviors that can deplete self-regulatory resources. In the present case, the inhibition was based on suppressing the tendency to use common words. The research we described earlier in the introduction indicates that many behaviors and actions can deplete self-regulatory resources. Our results suggest that learning RD categories also relies on some degree of cognitive inhibition and self-regulation. This claim is supported by earlier research showing deficits in RD category acquisition among populations with lower levels of inhibitory control (Minda et al., 2008; Huang-Pollock et al., 2011; Rabi and Minda, 2014; van Bers et al., 2014). This claim is also supported by past research showing reductions in RD category learning performance when performing concurrent tasks that require some degree of inhibition, switching, and self-regulation (Zeithamova and Maddox, 2006; Miles and Minda, 2011; Miles et al., 2014). Recent cognitive neuroscience research has suggested a correlation between being in a state of ego depletion and activation in the prefrontal cortex, which is the region most closely associated with the verbal/explicit system of COVIS (Friese et al., 2013).

The idea that inhibitory processes and executive functions are critical for certain types of tasks has implications for education. For example, our earlier research suggests that younger children with shallower pools of inhibitory resources perform less well on RD tasks (Minda et al., 2008; Rabi and Minda, 2014). This indicates that some children might not be well served by being asked to regulate their behavior prior to learning certain kinds of material. For example, consider a young child who wants to continually get out of his or her seat and wander around the classroom. This is disruptive, so the child may work very hard to regulate his or her behavior. But this regulation may come at a cost of reducing academic performance on subsequent learning opportunities or tests. Beyond this somewhat overt example, inhibitory processes may have more subtle effects on learning. For example, one recent study by Kaminski and Sloutsky (2013) examined the interpretation of bar graphs by six and eight-year-old children. Younger children had difficulty when the bar graphs contained extraneous information, like pictorial symbols. The researchers reasoned that it was more difficult for younger children to inhibit their attention to these extraneous details. Younger children had less difficulty with plain bar graphs. Older children did not suffer the same interference effects.

In summary, our results mesh well with many other studies suggesting that self-regulatory behavior and cognitive inhibition are a crucial component of complex learning behavior in general and of category learning behavior specifically. Our research also suggests that these resources can be depleted as a function of performing cognitively difficult tasks. This depletion can have detrimental effects on subsequent performance.

### CONTEXTUAL EFFECTS ON CATEGORY LEARNING

The results of our present study in many ways provide an interesting parallel to earlier studies in our lab investigating the effects of mood on category learning (Nadler et al., 2010), as well as the effects from other labs investigating the role of motivation on category learning (Maddox et al., 2006). With respect to mood, Nadler et al. (2010) demonstrated that when subjects were put in a positive mood, it significantly improved their performance on a RD category learning task, very much like the one we used here. Taken together, this suggests that for the same category task, 10 min of a self-regulatory task reduces performance significantly, and approximately 10 min of positive mood induction enhances performance. There are two speculative conclusions to be drawn from this. One is that these contextual effects can be strong, and can be brought about with even 10 min of engagement in the task. A second conclusion is that one might imagine that tasks like mood induction could be an antidote to the self-regulatory depletion effect. In other words, perhaps 10 min of positive mood could replenish the well of self-regulatory resources. In fact, Tice et al. (2007) found this to be the case and we expect their results to extend to the present category learning paradigm.

### MORE RESEARCH IS NEEDED

The suggestion that a positive mood seems to have the opposite effect on performance as the self-regulatory depletion task, suggests that other contextual factors which have a similar role as positive mood might be worth examining as well. For example, some research has indicated that ambient noise may improve performance on subsequent measures of cognitive flexibility, abstraction, and creativity (Mehta et al., 2012). These behaviors have also been linked to positive mood, so one possibility is that any contextual manipulations that have a calming effect could also serve to replenish cognitive resources and reduce or eliminate the ego depletion effect on category learning.

We can further speculate that additional research might show how the ego depletion effect can be mediated. For example, earlier research suggested that something as simple as additional glucose can reduce or remove the ego depletion effect by replenishing the levels of glucose in the blood (Gailliot et al., 2007). Consequently, one might carry out a study in which subjects are asked to complete the ego depletion task, where half are given a beverage with glucose, and the other half are given a beverage with no glucose. In this case we predict that the deleterious effect of the ego depletion condition would disappear on subsequent RD learning tasks.

## CONCLUSION

The results of our study suggest an interesting correspondence between ego depletion and the operation of the explicit/verbal system in category learning. Performing cognitively demanding tasks seems to reduce the ability of the explicit system to engage in the hypothesis testing necessary to learn new RD categories. The same cognitively demanding task does not seem to have an effect on the operations of the implicit/procedural category learning system. We think our results have implications for models of category learning by further clarifying the functionality of the two systems in CoVIS. We think our results also have implications for the creation of ideal learning environments and educational settings. Working hard to focus on a task, or to engage in multitasking, or to screen out distractions can take its toll. Sometimes, the mind needs to rest.

## Conflict of Interest Statement

The authors declare that the research was conducted in the absence of any commercial or financial relationships that could be construed as a potential conflict of interest.

## Footnotes

1 One participant made four errors and the other two participants made over 10 errors. Given that the stories participants wrote in the ego depletion condition were not very long to begin with, making four or more errors was considered substantial.

2 The same results held when we analyzed the data from all of the participants (i.e., including the data from the three participants originally excluded from analysis due to a high number of errors on the story writing task).
